# Calories, carbohydrates, and cancer therapy with radiation: exploiting the five R’s through dietary manipulation

**DOI:** 10.1007/s10555-014-9495-3

**Published:** 2014-01-17

**Authors:** Rainer J. Klement, Colin E. Champ

**Affiliations:** 1 Department of Radiotherapy and Radiation Oncology, Leopoldina Hospital Schweinfurt, Gustav-Adolf-Straße 8, 97422 Schweinfurt, Germany; 2 Department of Radiation Oncology, University of Pittsburgh Cancer Institute, Pittsburgh, PA USA

**Keywords:** Calorie restriction, Ketogenic diet, Metabolism, Radiotherapy

## Abstract

Aggressive tumors typically demonstrate a high glycolytic rate, which results in resistance to radiation therapy and cancer progression *via* several molecular and physiologic mechanisms. Intriguingly, many of these mechanisms utilize the same molecular pathways that are altered through calorie and/or carbohydrate restriction. Furthermore, poorer prognosis in cancer patients who display a glycolytic phenotype characterized by metabolic alterations, such as obesity and diabetes, is now well established, providing another link between metabolic pathways and cancer progression. We review the possible roles for calorie restriction (CR) and very low carbohydrate ketogenic diets (KDs) in modulating the five R’s of radiotherapy to improve the therapeutic window between tumor control and normal tissue complication probability. Important mechanisms we discuss include (1) improved DNA repair in normal, but not tumor cells; (2) inhibition of tumor cell repopulation through modulation of the PI3K–Akt–mTORC1 pathway downstream of insulin and IGF1; (3) redistribution of normal cells into more radioresistant phases of the cell cycle; (4) normalization of the tumor vasculature by targeting hypoxia-inducible factor-1α downstream of the PI3K–Akt–mTOR pathway; (5) increasing the intrinsic radioresistance of normal cells through ketone bodies but decreasing that of tumor cells by targeting glycolysis. These mechanisms are discussed in the framework of animal and human studies, taking into account the commonalities and differences between CR and KDs. We conclude that CR and KDs may act synergistically with radiation therapy for the treatment of cancer patients and provide some guidelines for implementing these dietary interventions into clinical practice.

## Background

Soon after the discovery of X-rays by Wilhelm Conrad Röntgen in 1895, ionizing radiation was utilized for cancer treatment. Today, it constitutes the standard of care for many cancer patients, along with surgery and chemotherapy. Recently, treatment outcomes have been improved in conjunction with a reduction in toxicity through technological innovations such as intensity modulated radiotherapy or stereotactic body radiotherapy. Despite these advancements, several cancer types continue to elude modern treatment techniques with radiation therapy (RT). Radioresistance of these tumors can be ascribed to two factors: environmental and intrinsic. The former include hypoxia, high lactate levels or the abundance of growth factors within the cellular microenvironment. Intrinsic factors include chronically activated proliferative, invasive, and antiapoptotic signaling pathways. A commonality between all of these factors appears to be the upregulation of glycolysis in cancer cells, resulting in the increased influx of glucose and excessive production of lactate regardless of partial oxygen pressure [1–3]. This phenomena was described nearly a century ago [4, 5], known as the Warburg effect, which affords cells both a high ATP generation and biomass synthesis [6]. It is the basic principle behind positron emission tomography (PET) with the glucose analog 2-(^18^F)fluoro-2-deoxy-d-glucose (FDG). PET studies have revealed that FDG uptake is inversely correlated with tumor control probability [7, 8] and overall survival [9], and areas with high FDG-PET have been suggested as targets for dose escalation with dose-painting RT [10].

**Fig. 1:**
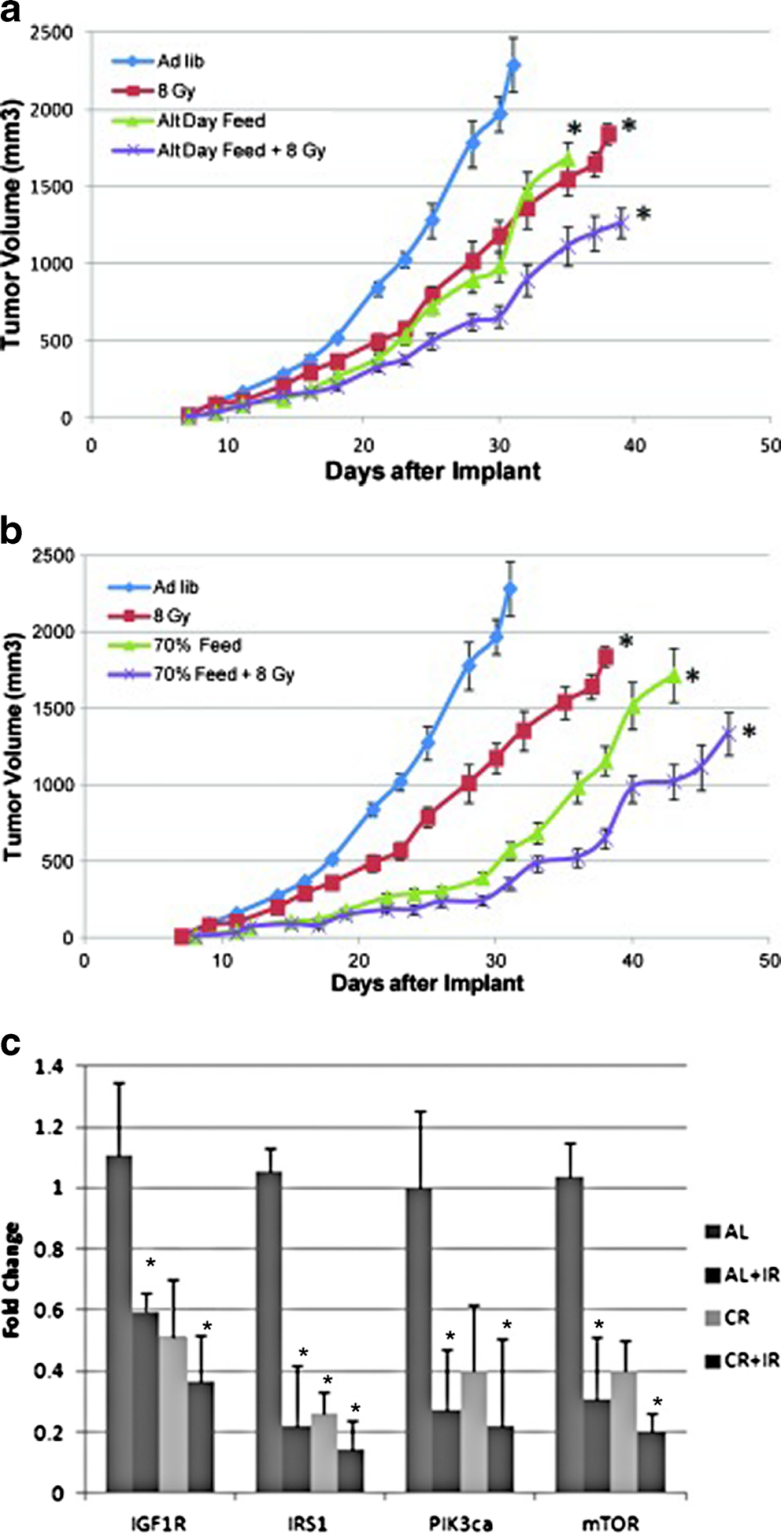
Nutrient deprivation *via* alternate day fasting (**a**) or overall caloric restriction (**b**) synergistically work with radiation therapy to significantly slow tumor growth and downregulate several key pathways (**c**). *AL* ad libitum feeding, *CR* calorie restriction (taken with permission from Saleh *et al.* [21])

The Warburg phenotype provides tumors an enhanced resistance against cytotoxic insults. In fact, work as early as 1933 has revealed that tumor cells have increased ability to resist radiation damage in the presence of elevated glucose [11]. However, this may come at the expense of metabolic flexibility. Hypoxia and genetic defects that chronically drive proliferation leave such tumors dependent on a steady supply of nutrients, especially glucose. Additionally, such tumors appear to benefit from pathological metabolic conditions of their host, in particular hyperglycemia, hyperinsulinemia, and elevated insulin-like growth factor (IGF)-1 levels [12, 13]. As a result, there has been recent enthusiasm towards metabolism-based therapies targeting whole-body metabolism, cellular kinases and glycolytic enzymes in order to sensitize these tumors to cytotoxic insults like RT [14–16]. As nutrition is a major modulator of global and cellular metabolism, it becomes apparent that nutritional interventions may impact cancer progression. In this context, metabolic targeting *via* calorie restriction (CR) has been described as a promising synergistic treatment option [17–19]. CR has consistently been shown to extend life span in organisms from yeast and worms to mice; furthermore, CR protects against age-related diseases like cancer [20]. While the beneficial effects of CR on whole-body metabolism, including improved insulin and glucose profiles, have been described for decades, recent research has revealed that, on a cellular level, CR affects the same molecular pathways as current biological agents proposed for targeting cancer metabolism. Recent data from our group [21] has revealed that caloric restriction in mice works synergistically with RT to target and downregulate several of these pathways and to slow tumor growth (Fig. 1). In humans, as discussed below, these molecular effects seem to be mediated mainly by the restriction of carbohydrates (CHOs) rather than total energy, which provides a rationale for the application of a very low carbohydrate, high-fat ketogenic diet (KD) in clinical practice [22]. Yet, the discussion of either CR or the KD as a low-cost and non-toxic treatment with multiple molecular targets is lacking in most discussions regarding metabolic targeting strategies.

The goal of this review is to therefore enhance the awareness for the potential benefits of CR and a KD as an adjunct to treatment for cancer patients during RT, and the strong preclinical data revealing that these modalities may enhance the efficacy of RT. Such benefits range from the cellular level to global metabolism, and underline the link between tumor cell metabolism and that of its host. Focus also lies on the commonalities and differences between these dietary modifications that should be considered when developing supplemental dietary treatment strategies.

## Calories or carbohydrates? Similar metabolic effects of calorie restriction and the ketogenic diet

CR is usually defined as a 30–50 % reduction in energy intake without malnutrition compared to *ad libitum* feeding. The caloric deficit can be induced either by intermittent fasting (IF), the extreme form of which is water-only short term fasting (STF), or chronic daily energy restriction (DER). However, as preclinical data is extrapolated to humans for clinical research design, it is important to point out that DER in mice corresponds to therapeutic STF in humans. Along these lines, fasting for 1 day in the mouse is roughly comparable to a 1-week water-only fast in a human [23].

**Fig. 2:**
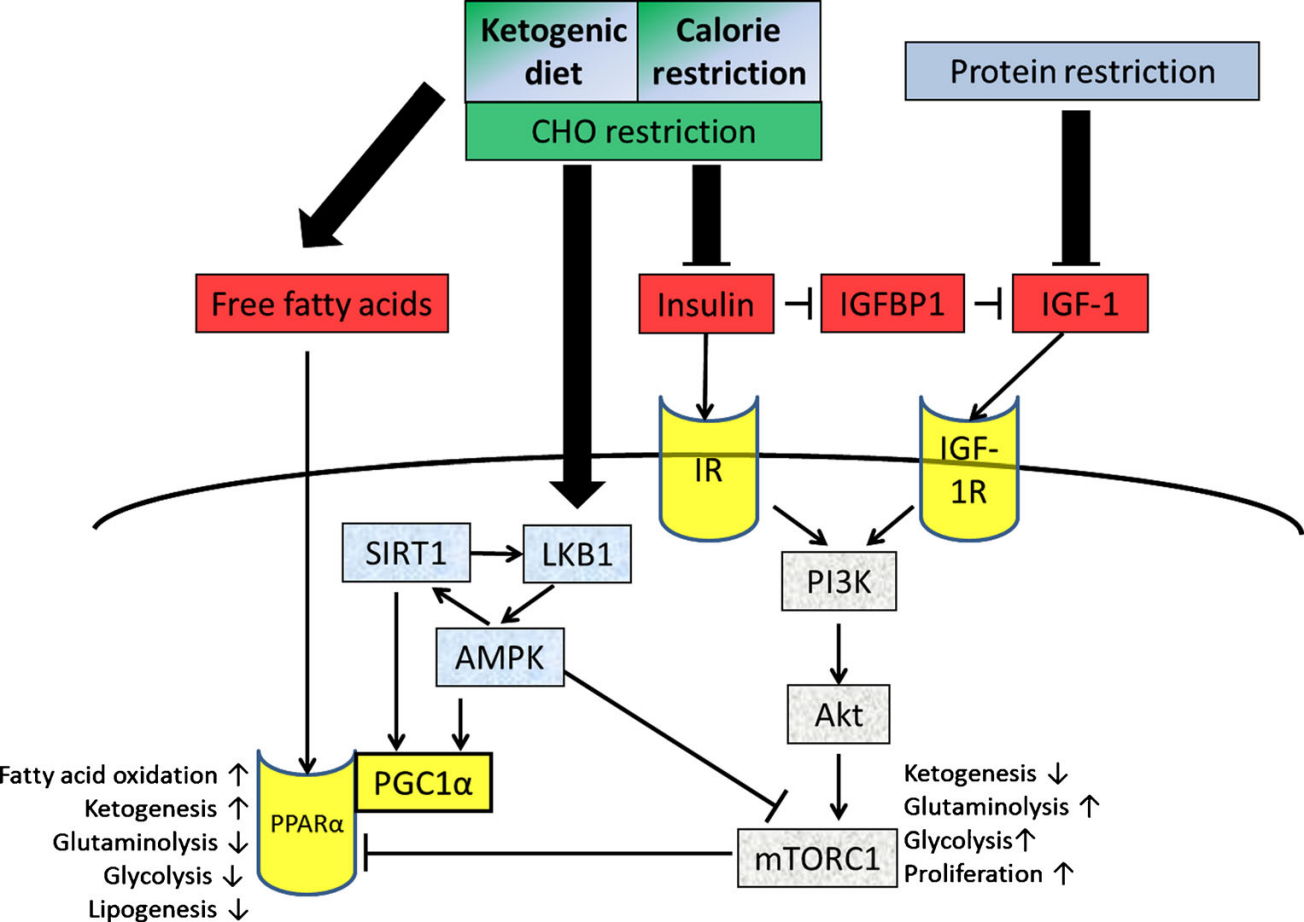
Calorie restriction (*CR*) and a ketogenic diet (*KD*) target the same molecular pathways that are also targeted individually by drugs to improve cancer treatment outcomes. *Arrows* indicate activation, *truncated lines* inhibition. Carbohydrate (*CHO*) restriction up-regulates fatty acid oxidation and ketogenesis (beneficial for normal tissues) and impairs glycolysis and glutaminolysis (detrimental to tumor cells). See Section 2 for more details

Protein restriction leading to a negative nitrogen balance has been shown to mediate the decrease of IGF-1 during CR [24, 25], explaining the significant decrease in IGF-1 after STF or the initiation phase of a KD [26], but not after several weeks of a KD [27] or long-term CR with adequate protein intake [25]. However, most other metabolic effects of CR appear to result from the accompanying restriction of CHOs [28]. KDs were actually developed in the 1920s as a method of mimicking fasting while avoiding malnourishment in the treatment of epilepsy [29]. The notion that KDs mimic the beneficial response to long-term fasting [30, 31] suggests the possibility to apply this dietary method to the oncological setting when weight loss must be avoided [22]. As displayed in Fig. 2, CHO restriction, whether through CR or a KD, decreases serum glucose and insulin levels, which increases lipolysis and leads to fatty acid-mediated activation of peroxisome proliferator-activated receptor α (PPARα). PPARα inhibits glycolysis and fatty acid synthesis, while promoting the transcription of enzymes that increase ketogenesis and mitochondrial and peroxisomal fatty acid oxidation [32]. The drop in insulin levels that accompanies the reduction in CHOs lowers the bioavailability of IGF-1 through increased transcription of IGF binding protein (IGFBP)-1 [33]. When insulin and free IGF-1 bind to their specific tyrosine kinase receptors they activate the phosphatidylinositol-3 kinase (PI3K)–Akt–mammalian target of rapamycin complex 1 (mTORC1) signaling pathway to promote many of the hallmarks of cancer including sustained proliferative signaling, resisting cell death and altered cellular metabolism including increased fermentation of glucose and glutamine [34]. mTORC1 downregulates ketogenesis through its inhibitory action on PPARα [35]. This action is counteracted during metabolic stress induced by CR or glucose withdrawal which decreases the intracellular ATP/AMP ratio and activates liver kinase B1 (LKB1)–adenosine monophosphate-activated protein kinase (AMPK) signaling. AMPK inhibits mTORC1 either directly through phosphorylation of the regulatory-associated protein of mTOR (Raptor) or indirectly by phosphorylating the mTOR inhibitor tuberous sclerosis complex protein-2 (TSC2). Increased lipid oxidation resulting from AMPK activation also increases the NAD^+^/NADH ratio thus amplifying the activity of the NAD^+^-dependent deacetylase silent mating type information regulation 2 homologue 1 (SIRT1) [36]. SIRT1 influences cellular lifespan and metabolism through epigenetic regulation of gene transcription and posttranslational protein modifications. Molecular targets of SIRT1 include LKB1 and peroxisome proliferator-activated receptor γ co-activator α (PGC1α), which is also activated through AMPK-mediated phosphorylation at Ser538 and Thr177 and cooperates with PPARα to induce mitochondrial biogenesis. This was demonstrated recently by Kitada *et al.* [37] in human skeletal muscle cells treated with serum obtained from four healthy obese subjects after a 25 % DER intervention lasting 7 weeks. Compared to treatment with serum obtained at baseline, there was a significant increase in AMPK, SIRT1, and PGC1α-mediated mitochondrial biogenesis. In addition, significantly higher levels of phospho-AMPK and phospho-SIRT1 were measured in peripheral blood mononuclear cells compared to baseline. Thus, CR and CHO withdrawal activate an energy sensing network consisting of AMPK, SIRT1, PPARα and PGC1α that promotes mitochondrial function and counteracts the insulin/IGF-1–PI3K–Akt–mTORC1 pathway. Studies by Draznin *et al.* [38] and Bergouignan *et al.* [39] suggest that CHO restriction alone, and even in the presence of caloric overconsumption, is sufficient for the activation of this network in human muscle cells, in line with the finding that AMPK is sensitive not only to the intracellular ATP/AMP ratio, but also to glycogen stores [40]. Studies have revealed increased phospho-AMPK levels in the liver, but not brain of rats fed a KD [41] and in the liver, but not epidermis or prostate of mice fed a 30 % CR diet [42], which implies tissue-dependent effects of CHO restriction on AMPK activation. Nonetheless, Akt and mTOR signaling were decreased by either the KD or CR in all of these tissue sites, again indicating the common effects of calorie and CHO restriction at the cellular level. Thus, CR and likely KDs target the same molecular pathways that are also targeted individually by drugs to improve cancer treatment outcomes, including Akt, mTOR, and AMPK (Fig. 2).

## How calorie and carbohydrate restriction may influence the response to radiotherapy

**Fig. 3:**
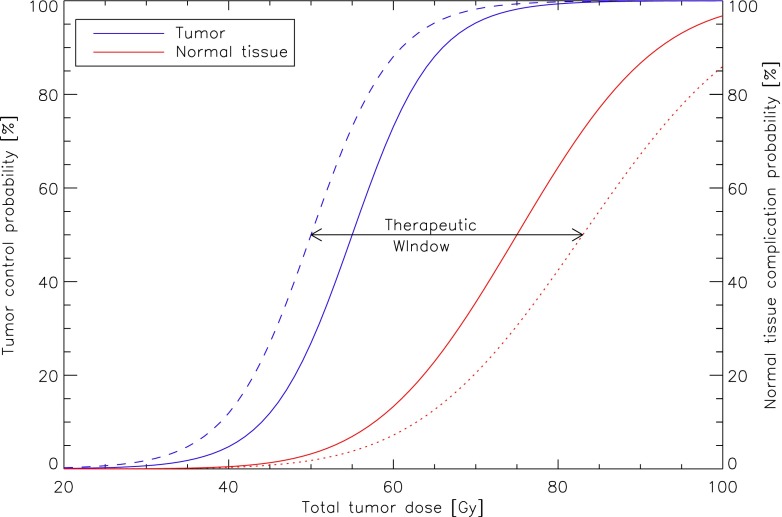
Illustration of a typical tumor control probability (*solid blue line*) and normal tissue complication probability (*red solid line*) curve as a function of total dose delivered to the tumor. We argue that CR and possibly a KD may increase the therapeutic window by favorably affecting both curves, *i.e.* a differential response between tumor and normal tissue

**Fig. 4:**
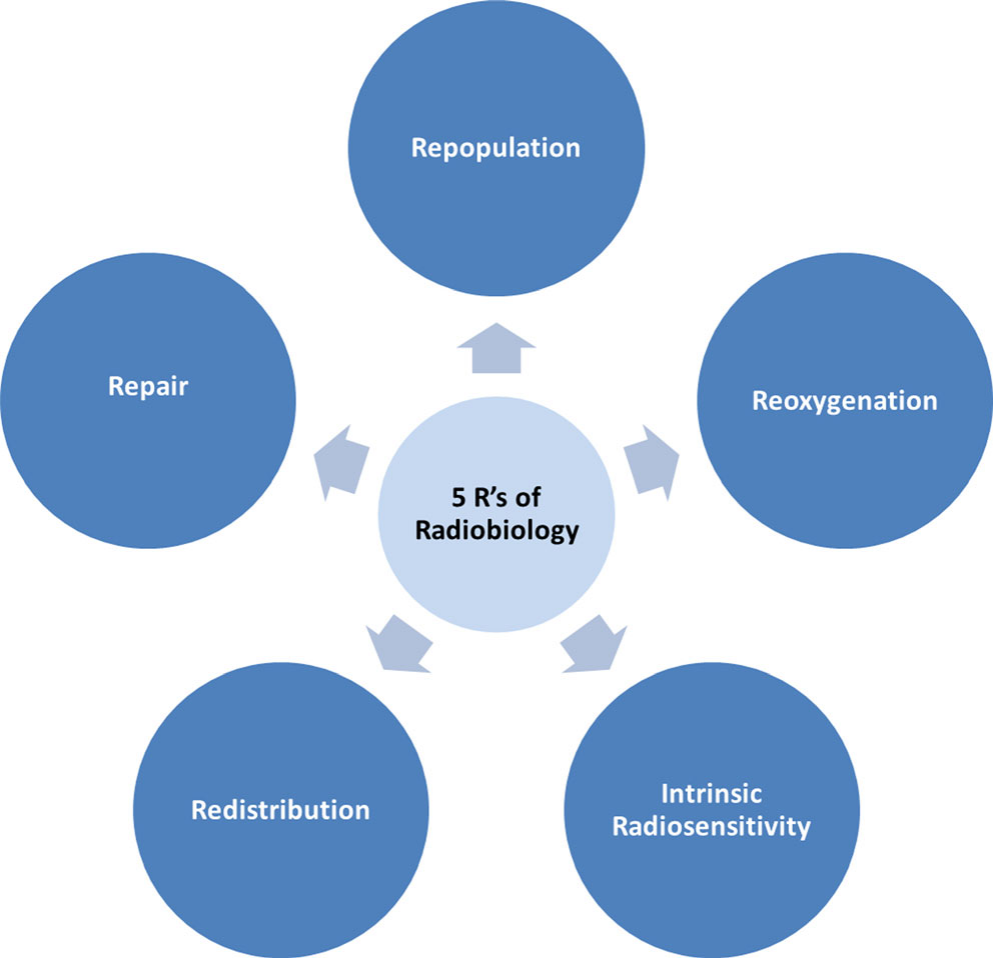
The five R’s of radiobiology

Most often, RT is applied in a fractionated fashion with typical doses per fraction in the range of 1.8–3 Gy. The biological rationale behind fractionated RT is based on exploiting the different responses between fast proliferating tumors and slowly proliferating normal tissue (Fig. 3). The factors underlying these responses are known as the “five R’s of radiobiology” (Fig. 4): Repair of sublethal DNA damage; Repopulation of the tumor; Redistribution of cells to different phases of the cell cycle; Reoxygenation of hypoxic tumor areas; and finally, intrinsic Radioresistance as suggested by Steel *et al.* [43]. The goal of RT is to utilize these factors in order to maximize the therapeutic window under the constraints of sufficiently large tumor control probability (TCP) and acceptable normal tissue complication probability (NTCP). Any additional intervention that increases TCP for a given dose while keeping NTCP constant, decreases NTCP at a given dose while keeping TCP constant, or both, will likely enhance treatment efficacy (Fig. 3). However, many pharmaceutical interventions do not increase the therapeutic window as they are often exceedingly unspecific and increase both TCP and NTCP at a given prescribed dose. In contrast, favorable treatment outcomes through a combination of CR [21, 44] or the KD [45, 46] with RT have been described in the literature. Data has demonstrated that CR or its pharmaceutical mimetic protects normal cells and sensitizes cancer cells to various common chemotherapeutic drugs; remarkably, this so-called differential stress resistance was observed across a wide range of normal and tumor cell lines, mouse strains and even humans [44, 47–51]. Apart from their direct relevance for patients undergoing simultaneous chemoradiation, these findings also suggest that CR or the KD may influence the five R’s of radiobiology (Fig. 4) in a manner that increases the therapeutic window.

### DNA damage repair

The interaction of ionizing radiation with molecules in tissue leads to the production of free electrons, leaving behind charged molecules with unpaired valence electrons called radicals. Radiolysis of water is the most frequent ionization event outside of the DNA and leads to the formation of reactive oxygen species (ROS) including the hydroxyl radical (OH^•^) and its reaction product with oxygen, hydrogen peroxide (H_2_O_2_). ROS are able to diffuse to and oxidize DNA at various sites including the sugar-phosphate backbone leading to single (SSBs) and double strand breaks (DSBs). While a single lesion can usually be repaired and is considered sublethal, accumulation of sublethal lesions with increasing dose can lead to their interaction and conversion to lethal lesions. Differences between tumors and normal tissues in the ability to repair sublethal damage are therefore an important rationale for fractionated RT.

Numerous studies suggest that CR enhances DNA repair of sublethal damage in normal tissues (reviewed in Ref. [52]), implying a role for CR in limiting toxicity to normal tissues during RT. Along these lines, CR may impact DSB repair, which is vital for cell survival between fractions [53]. The repair of DSBs is achieved by two different mechanisms known as non-homologous end joining (NHEJ) and homologous recombination repair (HRR). During NHEJ, the DSB ends are quickly recognized and bound by the Ku protein which subsequently recruits the catalytic subunit of the DNA-dependent protein kinase (DNA-PKcs) to form the DNA-PK holoenzyme. Binding to DNA triggers the kinase activity of DNA-PK which recruits and activates other proteins in order to clean and rejoin the DNA ends. Final ligation is carried out by the interaction of the XRCC4, ligase IV and XLF proteins. Although it can utilize short homologous sequences of up to 4 bp when possible, NHEJ does not necessarily conserve DNA sequences and is considered error-prone. In contrast, HRR is an error-free repair mechanism which requires DNA homology. It is therefore mostly efficient during and shortly after DNA replication in late S and G2 phases of the cell cycle when sister chromatids are available. It follows that HRR is an important pathway against DSB-induced lethality in fast proliferating tumors.

A dose of 1 Gy photon irradiation yields approximately 1.000 SSBs and 40 DSBs in a cell’s nucleus [54], a number that can be greatly enhanced through the combination with chemotherapeutic drugs. As noted previously, differential stress resistance between normal tissue and tumor cells has been observed when STF was combined with chemotherapy [44, 47–51]. STF likely selectively improves DSB repair in normal but not cancer cells. In the lung, liver, spleen, and kidney of aging rats, CR attenuated the decline of NHEJ activity [55]; this coincided with increased levels of XRCC4 in these tissues. Other NHEJ proteins like XLF and Ku may be upregulated by CR in a tissue-dependent manner [55, 56]. Like other DNA stress response genes, XRCC4 appears to be a target of the forkhead box O (FOXO) transcription factor family [55], which has been implicated with the antitumoral effects of CR [57]. FOXO-mediated transcription of stress response proteins is positively regulated by deacetylation through SIRT1, while phosphorylation through Akt leads to its nuclear exclusion and degradation. Furthermore, upon radiation-induced DNA damage SIRT1 binds to and deacetylates the repair protein Ku70, which enhances the efficacy of DSB repair [58]. Thus, CR and possibly a KD may positively affect NHEJ in normal tissue by increasing SIRT1 activity and decreasing insulin/IGF-1–PI3K–Akt signaling (Fig. 2). These protection mechanisms are likely defective in tumor cells with self-sufficiency in growth signals and constitutively activated PI3K–Akt pathway [50, 59].

Contrary to this, it is possible that CR impairs DSB repair in tumor cells and thus contributes to increased cell death. Chen *et al.* showed that mTOR inhibition through rapamycin or everolimus impairs both HRR and NHEJ in MCF7 breast cancer cells, without significant alterations in several important DNA repair proteins [60]. Importantly, a dose-dependent effect of CR on mTOR inhibition mediated by AMPK was also observed in a rat model of breast cancer [61], suggesting that fasting might have similarly negative effects on DNA repair capacity in mammary tumors as rapamycin. Song *et al.* incubated mouse fibrosarcoma cells with 5 mM metformin for 24 h before and after irradiation [62]. The treated cells exhibited a steeper survival curve with a narrower shoulder, indicating increased accumulation of sublethal lesions at a given dose and suggesting impaired DNA repair.

In summary, CR has been shown to enhance various DNA repair mechanisms in normal tissues including HRR and NHEJ, which are essential for RT-induced DSB repair. In contrast, repair capacity in cancer cells may be left unaffected or even attenuated through CR. The differential stress resistance between normal and cancerous cells to chemotherapeutic drugs seems to be mediated at least in part by decreased glucose and free IGF-1 levels [47, 50]; it could therefore be speculated that the KD might achieve similar effects, although this would have to be investigated in future studies.

### Repopulation of the tumor cells

Repopulation, *i.e.*, the cell proliferation occurring during the course of fractionated RT, occurs in both tumors and normal tissue, and provides the biological rationale behind altered fractionation schedules in certain cancer types such as accelerated fractionation in head and neck squamous cell carcinoma or hypofractionation in non-small cell lung cancer [63]. Such cancers respond to RT with an increase in tumor doubling times and hence accelerated proliferation during extended treatment times, therefore decreasing the TCP. Radiobiological modeling suggests that any strategy that delays the onset and/or decreases the rate of tumor repopulation could increase TCP at a given NTCP or decrease the late responding NTCP through the application of smaller doses in the presence of a larger number of fractions without impairing TCP [64]. Figure 3 demonstrates this effect qualitatively if one assumes the solid blue curve to be the TCP with accelerated repopulation starting within the treatment period. The dashed blue curve would indicate the lack of this effect, *i.e.*, a delay in the onset of accelerated repopulation to a time point after finishing the treatment. A quantitative calculation for non-small cell lung cancer based on the linear-quadratic formalism was performed by Fowler *et al.* [65], demonstrating that the TCP would be roughly doubled for a typical dose of 70 Gy given in 2-Gy fractions if accelerated repopulation of the tumor could be delayed long enough.

CR in rodents reduces IGF-1/insulin–PI3K–Akt–mTor signaling which has been shown to be correlated with significant tumor growth delay [21]. CHO restriction in patients with advanced cancer has also revealed downregulation of this pathway [66]. The causal and important role of this pathway in promoting tumor progression is exemplified by the fact that CR combined with IGF-1 administration [67] or constitutive PI3K activation through genetic mutations [59] rescues tumors from growth inhibition induced by CR. We recently reviewed the large number of animal studies showing the potential of CR [19] and KDs [22] to delay and retard tumor growth and even metastasis, often without additional treatment. CR in mice is able to slow tumor growth by 50–80 % though it is important to note that the majority of these studies reduced CHO within the diet and replaced it with fat. It still remains unclear if and to what extend these findings translate to humans, the more so as available data suffer from small sample sizes. A retrospective analysis of five patients with tuberous sclerosis complex yielded mixed results concerning tumor progression during a KD and in no case tumor regression was achieved [68]. Other data are more supportive for targeting tumor cell proliferation through CHO restriction. Rossi-Fanelli *et al.* [69] showed that a high-fat diet (80 % non-nitrogenous calories from fat) inhibited tumor cell proliferation while a high-dextrose diet (100 % non-nitrogenous calories from dextrose) increased proliferation over 14 days in patients with gastrointestinal cancers, though patient numbers were too small to reach statistical significance. Diets were administered parenterally and cell proliferation was assessed using thymidine labeling index on tumor samples, which measures the fraction of cells in the S phase as a proxy for *de novo* DNA synthesis. Zuccoli *et al.* reported on a female patient with GBM undergoing two therapeutic fasts followed by a KD restricted to 600 kcal/day and concomitant RT and temozolomide treatment [70]. This intervention stopped tumor growth completely as judged by MRI and PET imaging, but tumor recurrence occurred 10 weeks after suspension of this diet.

Fast proliferating cancer cells rely on a high glycolytic rate in order to shuffle phosphometabolites into the pentose phosphate pathway for biosynthesis of nucleic acids and lipids. Activation of PPARα by KD or CR promotes ketosis and inhibits glycolysis, therefore abating proliferation in tumor cells. In normal cells, abundant acetyl-CoA from the breakdown of ketone bodies and fatty acids inhibits glycolysis to ensure stable ATP levels; tumor cells which often have dysfunction mitochondria lack this flexibility and quickly die when confronted with glucose withdrawal [71–76]. This was exemplified in a study by Fine and colleagues [77], revealing that overexpression of uncoupling protein (UCP) 2, a common defect in tumor mitochondria, rendered these cells vulnerable to treatment with the ketone body acetoacetate [77]. In these cells, the decrease in glycolytic ATP production cannot be compensated by oxidative phosphorylation, leading to ATP depletion and cell growth inhibition. FDG-PET studies in cancer patients on a KD confirmed that CHO restriction with subsequent insulin inhibition and ketosis inhibits tumor glycolysis *in vivo* [66, 70, 78]. The importance of ketone bodies was thereby demonstrated by Fine and co-workers [66] who found a statistically significant correlation between the level of ketosis and partial remission or stable disease on PET scans after a 4-week KD in nine patients with prior rapid disease progression.

In conclusion, CR and KDs have shown significant inhibitory effects on tumor growth in animal studies which would predict a left-shift of the TCP curve (Fig. 3). Based on mechanistic insights that the IGF-1/insulin–PI3K–Akt–mTORC1 pathway and glycolysis play a key role for tumor cell proliferation and supported by positive evidence from small patient studies we hypothesize that CR and KDs could be used as supportive strategies to target tumor cell repopulation during RT.

### Redistribution in the cell cycle

Normal cells interrupt typical cell cycling after exposure to ionizing radiation in order to allow for enough time for DNA repair, or in case of extreme or irreparable damage, prepare for cell death or senescence. Transition from one phase of the cycle into the other is regulated by a family of kinases known as cyclin-dependent kinases (CDKs), whose activity is regulated through three mechanisms: (1) association with phase-dependent proteins called cyclins; (2) phosphorylation and de-phosphorylation; (3) inhibition by CDK inhibitors such as p21. Cells are most sensitive to DNA damage during replication and mitosis, *i.e.*, the S and M phases of the cycle, respectively. Therefore, phases preceding mitosis utilize a variety of molecular pathways known as checkpoints to ensure that necessary steps for a phase have been completed and no severe DNA damage has gone unrepaired. In tumor cells, checkpoints are often overridden by oncogenic activation of proliferative signaling *via* PI3K-Akt [79, 80] and/or loss-of-function of gatekeeper genes like *TP53*. It follows that with increasing RT fraction number, ionizing radiation leads to a decreasing fraction of normal cells in sensitive S and M phases while tumor cells are mostly unaffected by redistribution.

With a mutation rate of more than 50 %, the transcription factor p53 is the most frequently mutated gene in tumors. Important transcriptional targets of p53 include p21 and the growth arrest and DNA-damage-inducible protein Gadd45a, two CDK inhibitors that promote G1 and G2 arrest, respectively. p53 is strongly connected to the Warburg phenotype [81] and provides a rationale for the use of cycle-dependent chemotherapy. p53 mutations disrupt cytochrome *c* function, thus decreasing respiration. This leads to compensatory fermentation or the Warburg effect which can be targeted by glucose restriction. Apontes *et al.* [82] showed that rapamycin and metformin acted synergistically to induce G1/G2 arrest and protect normal cells under both normal and low glucose conditions against the mitotic inhibitor nocodazole, a drug causing lethal mitotic arrest; in contrast, the same treatment did not protect MDA-MB-231 breast cancer cells expressing mutant p53, and even was toxic under low glucose conditions. In addition to p53, other frequent mutations in cancer cells are responsible for constitutive activation of the PI3K–Akt pathway. These cells are able to overcome both the G1/S and G2/M checkpoints normally induced by DNA damage, and continue to divide [79, 80]. However, such cells may selectively be targeted by glucose withdrawal. Shim *et al.* [72] showed that c-Myc transformed cells underwent apoptosis upon glucose restriction, while normal cells remained intact in G0/G1 cell cycle arrest. Glucose restriction was also shown to exert opposite epigenetic effects upon human telomerase reverse transcriptase and the cell cycle regulator p16 between immortalized and normal fetal lung fibroblasts, such that the former underwent apoptosis while the latter responded with an extension of lifespan [74]. The same authors later identified SIRT1 as a key regulator of this mitigation of p16 in normal cells [83]. Paradoxically, fasting seems to stimulate the translation of genes involved in growth and proliferation and to further increase phosphorylation of Akt in oncogene-activated cells [51]. However, though this may appear to be “adding fuel to the fire” and driving tumor growth, it also demands increased energy production which eventually leads to an increase in ROS and cell death under low nutrient and growth factor conditions [51, 73, 76].

Conversely, in normal cells, the decrease of mitogenic stimuli induced by CR and perhaps to a lesser extent, the KD favors redistribution into a non-dividing state in order to preserve and redistribute energy for cellular protection mechanisms [50]. This finding can be exploited clinically by having patients fast prior to each RT session and/or chemotherapy cycle [48, 49]. Safdie *et al.* reported that fasting before and/or after chemotherapy decreased symptoms of weakness and fatigue, while reducing gastrointestinal side effects when compared to a normal diet in six cancer patients undergoing a median of four cycles of chemotherapy [48]. In C57BL/6J mice, CR upregulated Gadd45a and p21 in a FOXO1-dependent manner [57]. However, tumors with FOXO inactivation due to hyperactive PI3K–Akt signaling would be unable to benefit from CR-induced cell cycle arrest under irradiation, providing a further opportunity to widen the therapeutic window.

In summary, CR arranges a redistribution of normal cells in the cell cycle, potentially protecting them from subsequent DNA damaging insults like RT. The situation in tumor cells seems quite contrary. Here, fasting seems to promote cell cycle progression, M phase accumulation and energy expenditure, in this way rendering such cells synthetically vulnerable to the combination of nutrient restriction with RT or chemotherapy.

### Reoxygenation

A major challenge for RT is the presence of hypoxic areas within solid tumors. The lack of oxygen molecules within these regions inhibits the formation of H_2_O_2_ from OH^•^, thus lessening the frequency and severity of DNA damage. A single fraction of irradiation preferentially kills the well-oxygenated cells, but reoxygenation of hypoxic areas occurs during fractionated treatment in part due to tumor shrinkage. Hypoxia facilitates DNA repair and leads to stabilization of the α-subunit of hypoxia-inducible factor (HIF)-1, a transcription factor that lies downstream of mTOR and upregulates glycolysis [84]. The Akt–mTOR pathway upregulates the translation of HIF-1α mRNA in a glucose- and reoxygenation-dependent manner after irradiation [85].

Tumors possess a heterogeneous network of abnormal blood vessels characterized by chaotic anatomical arrangement, dead ends, and increased leakiness which leads to increased interstitial fluid pressure [86]. This results in areas with both chronic and acute hypoxia, the former occurring where oxygen supply is limited by diffusion from proximal blood vessels, and the latter where perfusion is transiently constricted. The abnormal vasculature is caused by an excess of pro-angiogenic signaling mainly due to vascular endothelial growth factor 2 (VEGF). VEGF is another target of HIF-1α, but its transcription is also increased through epigenetic modulation by inflammatory cytokines, growth factors, and sex hormones. Contrary to what may be expected from inhibiting VEGF and therefore new blood vessel formation, evidence has accumulated supporting the hypothesis that anti-VEGF therapy actually decreases hypoxia and facilitates the delivery of chemotherapeutic drugs to cancer cells by normalizing the vasculature which in turn normalizes the microenvironment [86].

Since VEGF is upregulated as a consequence of Akt–mTOR–HIF-1α signaling, any strategy that inhibits this pathway can be hypothesized to lower VEGF expression and tumor progression. Mukherjee, Seyfried, and colleagues have reported that CR downregulates VEGF and normalizes vascularization across a range of several rodent and human prostate and brain tumors [87–89]. In the CT-2A mouse astrocytoma, CR increased the perivascular cell coverage of blood vessels, insinuating decreased leakiness, less interstitial fluid pressure, and better drug delivery to the tumor [89].

Hyperbaric oxygen therapy (HBOT) is another approach to overcome hypoxia. The principle of HBOT encompasses breathing hyperbaric oxygen during irradiation in order to oxygenate and radiosensitize hypoxic cancer cells. A recent Cochrane review concluded that HBOT combined with RT may improve local control in head and neck and cervical cancers, but at the expense of significant adverse effects [90]. Recently, Poff *et al.* evaluated the combination of HBOT with a KD in the murine VM-M3 model of metastatic cancer which closely mimics several aggressive human cancers [91]. Interestingly, despite *ad libitum* feeding, mice on the KD lost about 10 % body weight, suggesting involuntary under-eating. While the KD alone increased mean survival time by 57 %, the combination of HBOT + KD increased survival time by 78 % compared to a standard diet. The translation of these results into clinical practice remains an open question. It can at least be hypothesized that ketone bodies might attenuate additional oxidative stress to normal tissues [92–94] but not cancer cells, which are unable to metabolize them [95–98].

### Intrinsic radiosensitivity

The Warburg effect seems to be a hallmark of radioresistant cancer cells. FDG uptake by tumors is a negative predictor of local control [7, 8] and survival [9], and is employed to guide the contouring of particularly radioresistant areas for dose escalation [10]. The high glycolytic rate appears to protect cancer cells from ROS-induced DNA damage by supplying large amounts of reducing equivalents like pyruvate, lactate, gluthatione, and NAD(P)H that scavenge ROS molecules [1]. Quantifying lactate *via* bioluminescence imaging in more than 1,000 individual xenografts of human HNSCC, Sattler and colleagues demonstrated that intra-tumoral lactate concentrations were significantly inversely correlated with tumor control after a 6-week RT schedule [99]. However, no such correlation was found for pyruvate, which can be explained by the fact that its concentration in tumors is much lower than that of lactate.

Ketone bodies and fatty acids inhibit glycolysis [32], which is why both fasting and the KD have the potential to target the antioxidative defense mechanisms outlined above. There is also evidence that due to dysfunctional mitochondrial electron transport chains, many cancer cells possess high steady state levels of ROS that quickly lead to cell death once glycolysis is impaired [46, 73]. On the other hand, oxidation of ketone bodies in peripheral tissue decreases the NADP^+^/NADPH ratio, which increases the amount of reduced gluthatione available for scavenging H_2_O_2_ [93]. This antioxidative property of ketone bodies would not benefit tumor cells which lack the necessary enzymes to metabolize them [95–98]. Furthermore, Shimazu and colleagues showed that beta-hydroxybutyrate (BHB) levels achievable after a several days fast or KD potently protected the kidneys of mice from oxidative stress measured by both protein carbonylation and lipid peroxidation [94]. The action of BHB and, to a lesser extent acetoacetate, was thereby related to their roles as class I and II histone deacetylase inhibitors, leading to histone acetylation with subsequent transcriptional activation of antioxidant genes like metallothionein and *Foxo3a*. Finally, the activation of the histone deacetylase SIRT1, which in humans occurs after CR [37], or more generally, CHO restriction [38, 39], has been shown to prevent H_2_O_2_-induced hyperacetylation of p53 in skeletal muscle cells, therefore protecting against oxidative stress in these tissues [37].

Cancer stem cells possess the highest intrinsic radiosensitivity and have been implicated in the failure to achieve local control, yet studies characterizing their metabolic phenotype are scarce. A recent study by Vlashi *et al.* suggests that such cells possess high metabolic flexibility and readily switch between glycolysis and oxidative phosphorylation if only one of these pathways is targeted [100]. This might indicate that—at least in the case of certain gliomas—CR or a KD alone is not sufficient to decrease ATP content and radioresistance in cancer stem cells.

## Clinical implementation

Dietary strategies that involve reducing food intake during cancer treatment leave the treating physician with trepidation as data has revealed that weight loss during treatment leads to poorer outcomes [101]. While significant weight loss from CR is a concern, fat loss in overweight patients during and after treatment may lead to an improved outcome as excessive adipose tissue in breast cancer patients may help fuel tumor cells [102]. However, recent data reveals that a CHO-restricted or KD may have a greater effect on attenuating metabolic factors associated with increased failure rates of RT, while avoiding the concern of both physician and patient in regards to severely restricting calories [103].

Most CR studies in animals employ a reduction in calories by 30 % or greater, and as discussed previously, such a restriction in mice is roughly comparable to a 1 week water-only fast in humans [23], both options that may not be reasonable for the cancer patient. This issue may be minimized through IF around RT treatments, as it results in less weight loss when used for periods of 2–3 months [51], similar to RT treatment times. Other pertinent issues include possible toxicity from CR, as chronic CR may decrease immune function [104] and impair wound healing [105], both issues for the post-operative and immunocompromised patient. Patients on a KD must also be closely monitored to ensure sufficient vitamins and nutrients are consumed for immunoprotection and adequate healing.

**Fig. 5:**
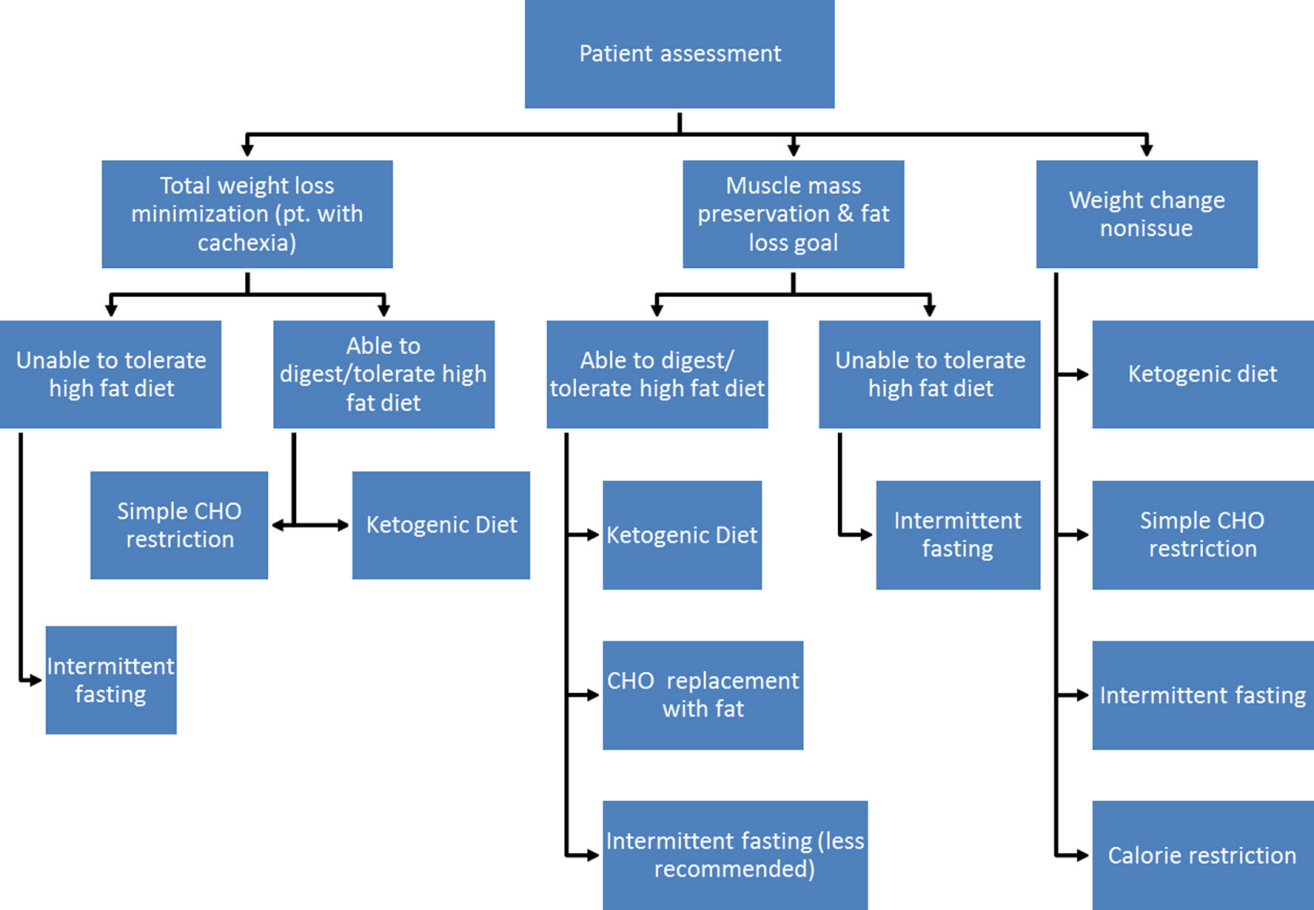
Proposed workflow of implementing dietary manipulation for cancer patients based on the results from an initial assessment

One of the first CR studies fasted conscientious objectors to WWII to 1,500 kcal/day while increasing their activity, leading to severe cachexia, malnourishment, and psychological detriment [106]. Such limits would be similar to those recommendations of 30 % or greater reduction in calories to achieve CR. While these patients were engaging in activity to increase their metabolic rate, this may not be dissimilar from the physiologic state resulting from a metabolically active tumor. The GBM patient on a KD reported by Zuccoli *et al.* was calorically restricted to 600 kcal/day [70]. Such limits on calories are not feasible in most oncologic settings, and more reasonable methods to achieve the metabolic effects of CR, without the potential of severe malnourishment and toxicity, include IF and CHO restriction [107]. Along these lines, preclinical data have revealed that the replacement of CHOs with fat may actually reduce cachexia [108], and clinical data have shown weight gain in pancreatic [109] and gastrointestinal [110] cancer patients with fat supplementation. However, patients must be assessed to ensure they can adequately tolerate a diet exceedingly high in fat (Fig. 5). We recently found that 5 weeks of a self-prescribed KD in healthy volunteers significantly increased bioelectrical phase angle [111], which is a proxy for muscle mass and a strong predictor of survival in cancer patients [112, 113]. Furthermore, randomized dietary studies in noncancer patients have revealed a significant decrease in blood glucose and the insulin pathway with a non-calorically but CHO-restricted diet *versus* a low-fat, calorically restricted diet [reviewed in Ref. 114]. Even caloric excess by 40 % in conjunction with CHO restriction appears to result in AMPK upregulation, pointing towards CHO and not calories as the prime target of dietary intervention [38].

## Conclusions

Dietary manipulation through CHO restriction, CR, and a KD may enhance the efficacy of radiation therapy by exploiting the five R’s of radiotherapy, while simultaneously reducing treatment-related toxicity. The treating physician, however, must weigh the benefits and risks of each dietary intervention, as each may be suitable in varying situations. While there is an ample amount of preclinical data, and clinical data continues to accumulate, further studies must take place comparing the different methods of dietary manipulation during radiation treatment and assessing their impact on tumor progression.
